# Copine proteins and derived bioactive peptides in tissue development and regeneration: from molecular mechanisms to biomaterial functionalization

**DOI:** 10.3389/fbioe.2026.1889849

**Published:** 2026-10-21

**Authors:** A. Tymińska, A. Kowalska, M. Rogińska, M. Pikuła

**Affiliations:** 1 Laboratory of Tissue Engineering and Regenerative Medicine, Department of Anatomy, Division of Embryology, Faculty of Medicine, Medical University of Gdańsk, Gdańsk, Poland; 2 Department of Biochemistry, University of Physical Education and Sport, Gdańsk, Poland

**Keywords:** angiogenesis, biomaterial functionalization, calcium-dependent signaling, CDP4 peptide, chondrogenesis, copine proteins, CPNE7, osteogenesis

## Abstract

The Copine (CPNE) family comprises evolutionarily conserved, calcium-dependent phospholipid-binding proteins (CPNE1-CPNE9) that function as intracellular membrane adapters and signaling modulators. In response to intracellular Ca^2+^ fluctuations, CPNE proteins translocate to the plasma membrane and recruit specific effector proteins, thereby integrating calcium dynamics with membrane remodeling, vesicular transport, and cell differentiation. While individual members display tissue-specific expression patterns-such as CPNE6 in hippocampal synaptic plasticity and CPNE7 in mesenchymal cell differentiation during odontogenesis-recent translational focus has centered on CPNE-derived bioactive peptides. Short synthetic sequences derived from the CPNE7 C2B-vWA junction region, specifically Cpne7-DP and CDP4, replicate key pro-regenerative functions of full - length proteins without requiring cell - surface receptor binding. When functionalized into biomaterial matrices (such as collagen gels and chitosan hydrogels via modified sequences UG27 and UG28), these peptides stimulate dentin tubule regeneration, osteogenic lineage commitment, biomineralization, and chondrocyte extracellular matrix deposition and angiogenesis. This review synthesizes current knowledge on CPNE structural biology, intracellular membrane translocation mechanisms, physiological tissue development, and oncological diagnostic markers.

## 1 Introduction

The Copine (CPNE) protein family comprises highly conserved, calcium-dependent phospholipid-binding proteins identified across eukaryotic organisms, ranging from unicellular protists (*Paramecium, Dictyostelium*) and plants (*Arabidopsis thaliana*) to nematodes and mammals (Creutz et al., 1998; Tomsig and Creutz, 2000; Tang et al., 2021). In mammals, nine distinct paralogs (CPNE1-CPNE9) have been characterized. Contrary to earlier general statements, the human *CPNE* genes are distributed across multiple chromosomes: *CPNE1* is localized to chromosome 20 (20q11.22), whereas *CPNE2* (chromosome 16 (16q13)) through *CPNE9* (chromosome 3 (3p25.3)) reside on distinct chromosomal loci across the genome (Khvotchev and Soloviev, 2024).

Evolutionarily, plant copines-such as BONZAI 1, 2, and 3 (BON1/2/3) in *A. thaliana*-play pivotal roles in balancing plant growth, development, and brassinosteroid signaling with disease resistance and immune responses (Hua et al., 2001; Yang et al., 2006; Wang Z. et al., 2011; Jing et al., 2024). This evolutionary conservation across kingdoms underscores the fundamental role of copines as adaptive modules that tune cellular responses to environmental and intracellular calcium signals.

In mammalian systems, CPNE proteins act as intracellular Ca^2+^ sensors and membrane adapters. Upon elevation of cytosolic Ca^2+^, copines translocate from the cytosol to the plasma membrane and intracellular organelles, where they modulate target protein localization, vesicular trafficking, cytoskeletal reorganization, and gene expression (Perestenko et al., 2010; Khvotchev and Soloviev, 2024).

## 2 Protein structure and domain architecture

Copine proteins have a modular domain architecture consisting of two C2 N-terminal domains (C2A and C2B), followed by a C-terminal domain that is structurally homologous to the von Willebrand factor type A (vWA) domain (Nalefski and Falke, 1996; Creutz et al., 1998; Tomsig and Creutz, 2000) (Figure 1).

**FIGURE 1:**
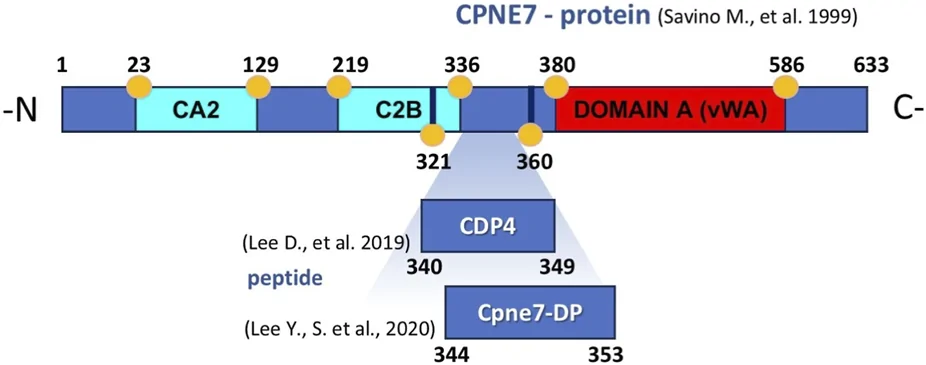
Domain architecture of CPNE7 showing the tandem C2A/C2B domains and the intracellular vWA domain. The peptides-origin regions (residues 340–353) is indicated at the C2B-vWA junction (Savino et al., 1999; Lee et al., 2019; 2020).

### 2.1 C2 domains and calcium-dependent membrane translocation

The C2 domains, which occur in a tandem arrangement (C2A and C2B), serve as the primary mechanism for calcium detection and phospholipid binding in copine. C2 domains interact directly with negatively charged cell membrane phospholipids (such as phosphatidylserine and phosphatidylinositol 4,5-bisphosphate) after coordinating Ca^2+^ ions within their structures (Nalefski and Falke, 1996; Tomsig and Creutz, 2000). Individual CPNE paralogs exhibit different affinities for Ca^2+^ and different translocation kinetics (Perestenko et al., 2010). Detailed structural mapping conducted by Perestenko et al. (2010) showed that while the C2 domains are responsible for the initial binding of calcium, a conserved sequence of 22 amino acids immediately following the C2B domain is absolutely essential for directing the protein toward the cell membrane and for its stable anchoring there.

### 2.2 The vWA domain

The intracellular domain of vWA is found in copine proteins and extracellular adhesion molecules (vWA/I). In integrins, it binds extracellular matrix (ECM) proteins via the MIDAS motif (metal-ion-dependent adhesion site, for example, Mg^2+^ or Mn^2+^) (Whittaker and Hynes, 2002), whereas the vWA domain of copines is strictly intracellular (Tomsig and Creutz, 2000; Tomsig et al., 2003) (Figure 1). The vWA domains of copine use coordination similar to that of MIDAS to recruit cytosolic target proteins, such as ubiquitin, 14-3-3γ, actin-binding proteins, and transcription factors, forming Ca^2+^-regulated, multiprotein signaling complexes on the inner layer of the cell membrane and on intracellular organelles. This intracellular role has been established by biochemical mapping of copine complexes and C-terminal vWA interactions (Tomsig et al., 2003; Khvotchev and Soloviev, 2024).

### 2.3 CPNE7 C2B-vWA junction

The CPNE7-derived peptides Cpne7-DP (KYKQKRRSYK, residues 344–353) and CDP4 (VNPKYKQKRR, residues 340–349) originate from the junction between the C2B domain and the C-terminal vWA domain (Lee et al., 2020; Tymińska et al., 2024) (Figure 1). This region lies within a conserved, positively charged stretch that contributes to Ca^2+^- regulated membrane association and protein recruitment. Although direct binding partners of this short segment have not been fully mapped, its high density of lysine and arginine residues confers cell-penetrating properties and facilitates interaction with negatively charged phospholipids and nucleic acids, providing a structural rationale for its use as a template for bioactive peptide design.

## 3 Cellular mechanisms and tissue development

### 3.1 Cpnes in the nervous system: Ca^2+^- dependent adaptors in synaptic and neuronal signaling

Copine proteins act as Ca^2+^- dependent membrane adaptors in neurons, tuning intracellular signaling rather than serving as primary instructive regulators of neurodevelopment. Among mammalian paralogs, *CPNE6* (chromosome 14 (14q11.2)) is the best-characterized neuronal member. It is strongly expressed in post-mitotic hippocampal and prefrontal cortex neurons (Reinhard et al., 2016; Khvotchev and Soloviev, 2024), with peak human expression occurring around age 5-later than in non-human primates-suggesting a developmental shift associated with prolonged synaptic maturation (Liu et al., 2012).

Upon NMDA receptor-mediated Ca^2+^ influx, CPNE6 translocates to dendritic spines, where it stabilizes the actin cytoskeleton through the Rac1-PAK-LIMK1-Cofilin pathway (Figure 2) (Reinhard et al., 2016). This mechanism supports long-term potentiation (LTP), learning, and memory consolidation. *Cpne6* knockout mice exhibit impaired LTP and deficits in hippocampus-dependent memory tasks (Shao et al., 2020; Li N. et al., 2025), underscoring its role as a Ca^2+^ sensor linking neuronal activity to structural plasticity.

**FIGURE 2:**
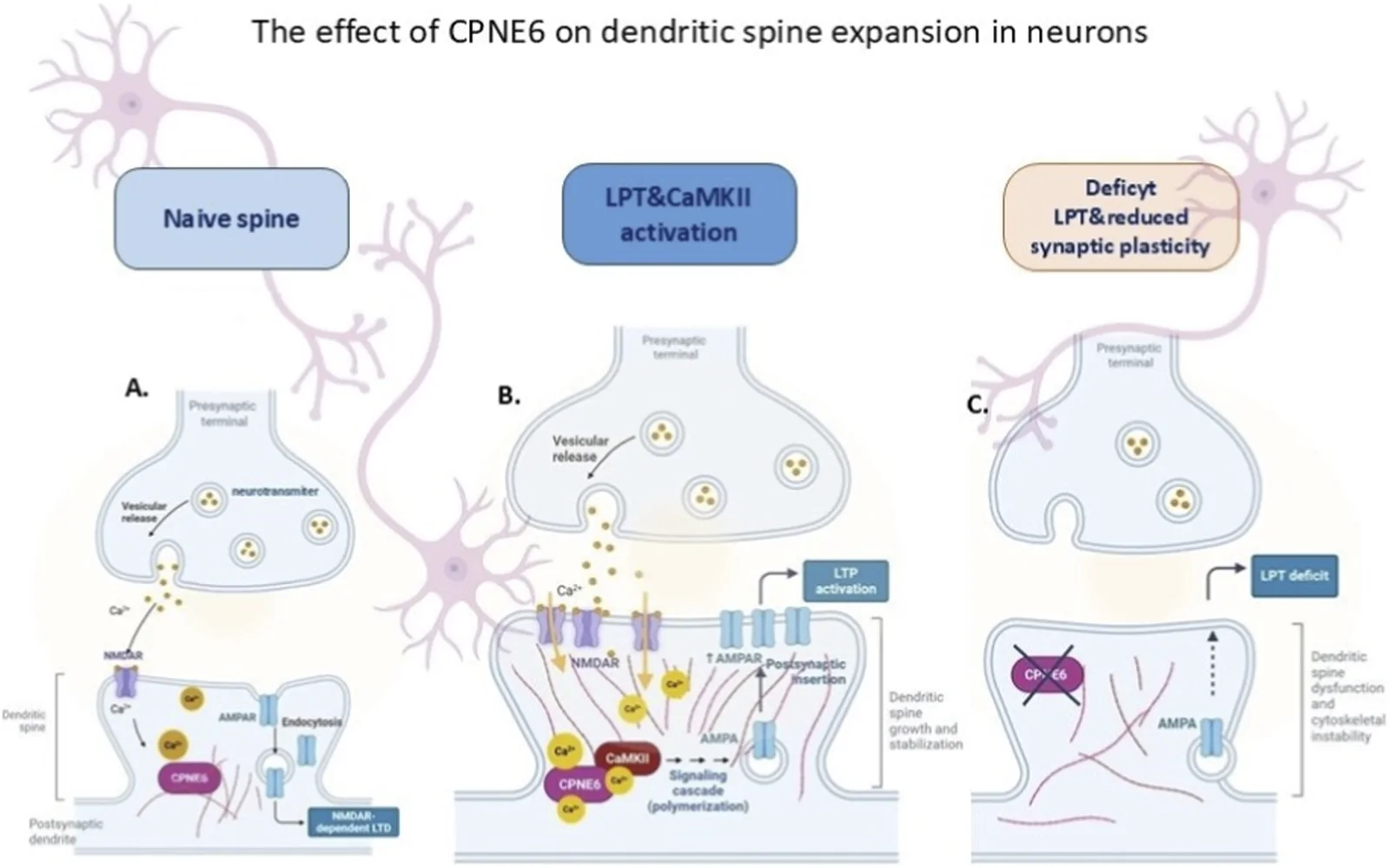
CPNE6 as a Ca^2^+ sensor regulating dendritic spine expansion through the Rac1–PAK–LIMK1–Cofilin pathway during long-term potentiation. **(A)** Naive spine–CPNE6 acts as a calcium sensor, facilitating the preparation of synaptic vesicles for neurotransmitter release. **(B)** LTP and CaMKII Activation–following Ca^2+^ influx via NMDA receptors, CPNE6 binds to CaMKII, leading to the insertion of AMPA receptors and stabilization of the spine structure. **(C)** Spine Expansion and LTP Deficit–the absence of CPNE6 (knockout) causes cytoskeletal instability, a reduced number of AMPA receptors, and an LTP deficit. Original figure by Biorender.

Other paralogs contribute to neuronal physiology in more context-dependent ways. CPNE1 regulates neural stem cell proliferation and differentiation through AKT-mTOR signaling (Kim et al., 2018), while CPNE3 participates in β-cell glucose uptake and insulin secretion (El-Huneidi et al., 2021) but also modulates neuronal cytoskeletal organization and vesicular trafficking (Tomsig et al., 2003; Goel et al., 2019). Current evidence supports the view that CPNEs act as contextual modulators of Ca^2+^-dependent membrane signaling rather than universal regulators of neurodevelopment.

### 3.2 CPNE7 in odontogenesis and ciliogenesis: a model of epithelial - mesenchymal signaling

CPNE7 provides the clearest mechanistic example of copine involvement in mammalian tissue development. During odontogenesis, preameloblast-secreted CPNE7 binds to cell-surface nucleolin (NCL) on dental pulp cells and is internalized through caveolae-mediated endocytosis (Figure 3) (Seo et al., 2017). After nuclear translocation, CPNE7 *-* encoded by the *CPNE7* gene on (chromosome 16 (16q24.3)) regulates the expression of *Kif3a* and *Ift88*, two core components of primary cilia. Enhanced ciliogenesis promotes the expression of dentin sialophosphoprotein (*Dspp*) and dentin matrix protein-1 (*Dmp1*), driving odontoblast differentiation and physiological dentin tubule formation (Oh et al., 2015; Seo et al., 2017; Lee et al., 2019).

**FIGURE 3:**
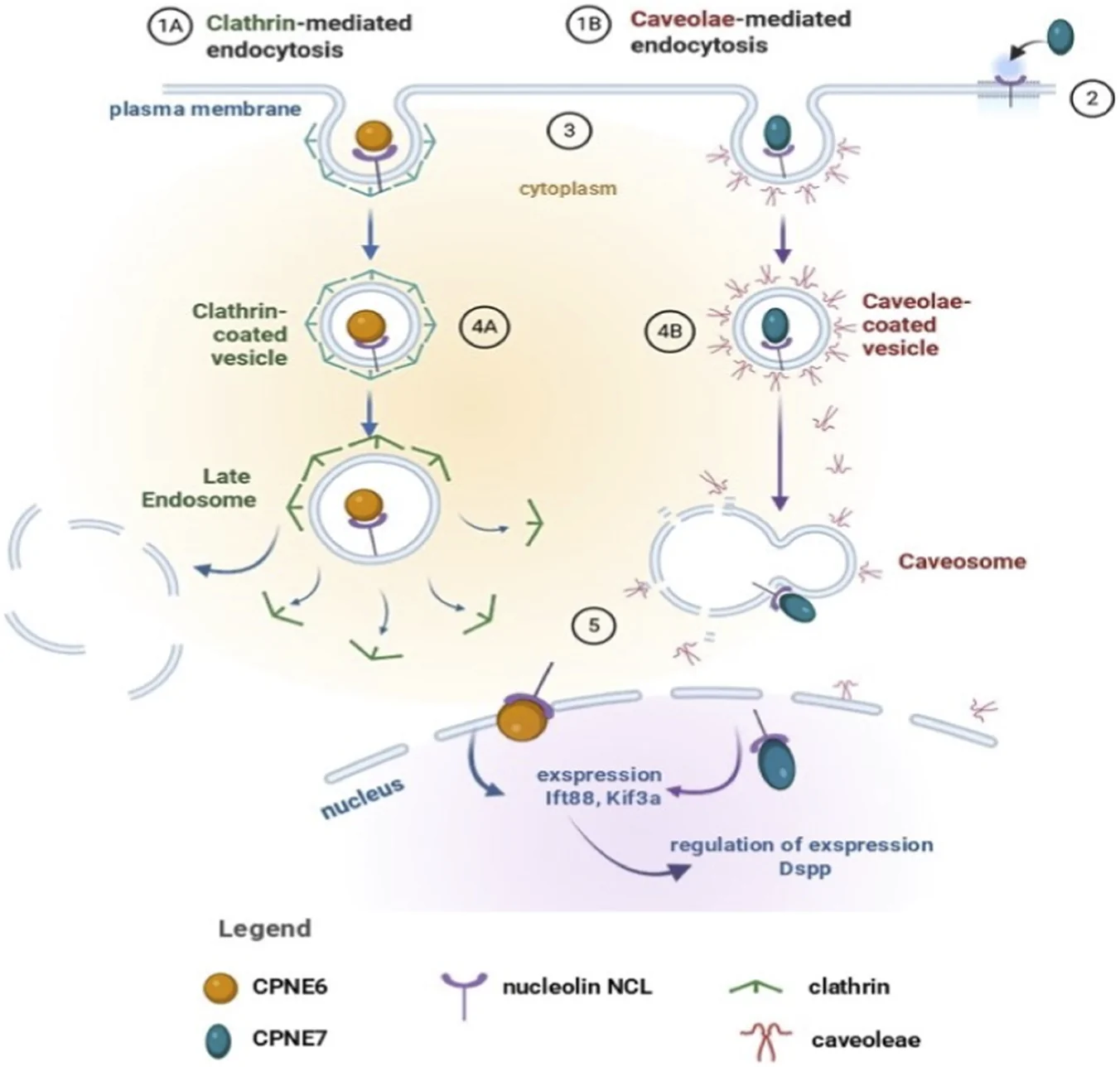
CPNE7 internalization via nucleolin and caveolae-mediated endocytosis, followed by nuclear translocation and induction of Kif3a/Ift88 expression during odontoblast differentiation.1A,B. Cpne7 binds to the nucleolin receptor on the cell surface. 2. Nucleolin forms a protein complex with Cpne6/7. 3. The complex is internalized into the cell via caveolin. 4A. A vesicle surrounded by a clathrin coat.4B. An endosomal caveolin vesicle forms in the cytoplasm. 5. The complex is translocated to the nucleus, expression of *Ift88* and *Kif3a* regulates the expression of dentin sialophosphoprotein (*Dspp*) (Seo et al., 2017). Original figure by Biorender.

This pathway illustrates how CPNE7 integrates Ca^2+^-dependent membrane association with transcriptional regulation during epithelial - mesenchymal interactions. CPNE7 does not act as a master regulator of odontogenesis; instead, it functions as a Ca^2+^-responsive adaptor that modulates ciliary signaling and lineage commitment. Its restricted expression pattern (fetal brain, thymus, testes, dental tissues) and defined receptor interaction make CPNE7 the most suitable CPNE paralog for translational peptide design (Cowland et al., 2003; Perestenko et al., 2010).

### 3.3 Plant copines and brassinosteroid signaling: evolutionary insights from BON1/2/3

Plant copines-particularly BONZAI 1, 2, and 3 (BON1/2/3) in *Arabidopsis thaliana-*provide a robust developmental model that highlights the conserved role of copines as Ca^2+^-dependent membrane adaptors. BON proteins regulate brassinosteroid receptor dynamics, balancing growth and immune responses (Hua et al., 2001; Yang et al., 2006; Wang Z. et al., 2011; Jing et al., 2024). Loss-of-function BON mutants exhibit impaired cell expansion, altered receptor trafficking, and heightened pathogen sensitivity, demonstrating how copines integrate environmental cues with intracellular Ca^2+^ signaling.

Recent work (Jing et al., 2024) shows that BON proteins modulate the trade-off between growth and immunity by controlling the localization and rotation of brassinosteroid receptors in the cell membrane. These findings highlight the system’s ability to perform the same function in different ways (functional redundancy) among plant copines and illustrate how a conserved domain architecture can support diverse biological outcomes across different kingdoms.

In the context of this review, plant copines strengthen the conceptual framework that CPNE proteins act as adaptive Ca^2+^-regulated modules, tuning membrane signaling rather than serving as primary developmental drivers. Their inclusion provides evolutionary grounding for CPNE7-derived peptide design and highlights the broader biological relevance of copine-mediated membrane regulation.

## 4 Dual roles of CPNE in regeneration and pathology

### 4.1 Oncogenic drivers vs. correlational biomarkers

The involvement of CPNE proteins in human malignancies illustrates their dual biological roles: while some paralogs act as causal oncogenic drivers, others serve primarily as correlative biomarkers without demonstrated mechanistic contribution.

CPNE1 is the most consistently validated oncogenic paralog. Its upregulation has been documented in clear cell renal cell carcinoma (ccRCC), non-small cell lung cancer (NSCLC), triple-negative breast cancer (TNBC), and colorectal cancer (Shao et al., 2020; Tang et al., 2021; Wang et al., 2021; Zhou et al., 2023). Functional studies demonstrate that CPNE1 promotes proliferation, radio-resistance, and chemoresistance through activation of the AKT-GLUT1/HK2 glycolytic pathway, establishing a causal role rather than a mere association. In ccRCC, CPNE1 expression correlates with tumor grade and invasiveness, and may be regulated by EGFR/STAT3 signaling (Zhou et al., 2023).

The CPNE3 protein exhibits strongly context-dependent behavior; the underlying *CPNE3* gene is localized to chromosome 8 (8q21.3). In breast, prostate, gastric, and lung cancers, CPNE3 enhances migration and metastasis via ErbB2, FAK, and PI3K - Akt signaling (Lin et al., 2018; Tang et al., 2021; Li et al., 2024). However, in glioblastoma, CPNE3 overexpression suppresses invasion and proliferation by inactivating FAK (Shi et al., 2021). This bidirectional behavior underscores that CPNE3 is not a universal oncogenic driver but a conditional modulator whose effects depend on cellular context, receptor repertoire, and microenvironmental cues.

The *CPNE8* gene (chromosome 12 (12q12)) is emerging as a potential oncogenic driver in gastric cancer, where it promotes metastasis by modulating focal adhesion kinase (FAK) and ERK signaling and activating fibroblasts within the tumor microenvironment (Zhang et al., 2022). Its role appears mechanistically stronger than that of CPNE5 or CPNE9 but still requires functional validation in additional cancer models.

In contrast, CPNE5 - encoded by the *CPNE5* gene chromosome 6 (6p21.3) - and CPNE9 function primarily as diagnostic or prognostic biomarkers. Elevated CPNE5 expression predicts longer survival in multiple myeloma (Skawran et al., 2008), while CPNE9 is associated with pancreatic cancer progression and glioblastoma prognosis (Zhu et al., 2016; Yang et al., 2018). These associations lack functional manipulation (knockdown/overexpression) and therefore should not be interpreted as causal.

The 2021 overview by Tang et al. provides a comprehensive synthesis of CPNE roles across cancers and emphasizes the need to distinguish correlative expression patterns from mechanistic evidence (Tang et al., 2021). Incorporating this distinction is essential when evaluating CPNE paralogs as potential therapeutic targets or when designing CPNE-derived peptides for regenerative applications.

### 4.2 Nucleolin targeting and off-target risks

Nucleolin (NCL) is a multifunctional protein expressed in the nucleus, cytoplasm, and-critically-on the cell surface of activated endothelial cells, immune cells, and many malignant tumors. Its involvement in CPNE7 uptake during odontogenesis (Seo et al., 2017) provides a mechanistic basis for CPNE7-derived peptide design but also raises important safety considerations.

The internalization of CPNE7 depends on nucleolin-mediated endocytosis, a pathway shared with several tumor-associated ligands, including lactoferrin, LPS, and endostatin (Bugler et al., 1982; Legrand et al., 2004; Wang Y. et al., 2011). Because nucleolin is upregulated in tumor vasculature and contributes to angiogenesis, proliferation, and immune modulation, any therapeutic strategy involving nucleolin-binding peptides must consider the possibility of off-target activation in occult or subclinical tumor microenvironments.

Short CPNE7-derived peptides such as Cpne7-DP bypass nucleolin and enter cells directly as cell-penetrating peptides (Lee et al., 2020), reducing receptor-specific risks. However, full-length CPNE7 and any engineered peptides retaining nucleolin affinity may theoretically stimulate endothelial proliferation or angiogenesis in tumor vasculature, enhance survival pathways in nucleolin-positive cancer cells, or alter immune cell activation in inflammatory or neoplastic tissues.

Given that CPNE1, CPNE3, and CPNE8 have documented oncogenic roles, and nucleolin itself is a therapeutic target in oncology, CPNE7-derived peptides must be evaluated with particular caution. Localized, biomaterial-based delivery (e.g., collagen or chitosan scaffolds) is therefore essential to restrict peptide exposure to the intended tissue defect and minimize systemic distribution.

Furthermore, nucleolin’s involvement in innate immunity and viral entry suggests additional off-target risks that should be assessed in preclinical models (Vester et al., 2021; Kitagawa et al., 2022; Pandey and Mishra, 2025), including effects on macrophage activation, endothelial barrier function, and inflammatory cytokine release.

## 5 CPNE7-derived peptides and biomaterial functionalization

### 5.1 Cpne7-DP and dentin regeneration

Cpne7-DP is a 10-amino-acid peptide (KYKQKRRSYK, residues 344–353 of CPNE7) derived from the C2B-vWA junction region. Unlike full-length CPNE7, which requires nucleolin-mediated endocytosis (Seo et al., 2017), Cpne7-DP behaves as a cell-penetrating peptide (CPP) capable of direct translocation across plasma membranes (Lee et al., 2020). This bypasses receptor-specific uptake and reduces nucleolin-related off-target risks.

*In vitro* and *in vivo* studies demonstrate that Cpne7-DP induces odontoblast differentiation by upregulating *Dmp1, Dspp*, and *Nestin*, promoting physiological tubular dentin formation and dentinal tubule occlusion (Lee et al., 2020). In rodent models, Cpne7-DP restores dentin structure and inhibits demineralization, suggesting potential utility in dentin hypersensitivity and regenerative endodontics. However, current evidence is limited to small animal models and short-term outcomes, and mechanistic pathways downstream of peptide internalization remain incompletely defined.

### 5.2 CDP4 structure - activity relationship and stability (SAR)

CDP4 is a 10-amino-acid peptide (VNPKYKQKRR, residues 340–349 of CPNE7) with a molecular weight of ∼1315 Da (Tymińska et al., 2024) (Figure 1). Its net positive charge (+5) arises from three Lysine and two Arginine residues, forming a highly basic C-terminal cluster (KYKQKRR) characteristic of CPPs (Ruczynski et al., 2015; Lee et al., 2019).

#### 5.2.1 Biophysical basis of CDP4 internalization

The internalization matrix of CDP4 is fundamentally dictated by its highly basic, cationic C-terminal tail (KQKRR), which serves as the primary engine for cellular uptake. Because the eukaryotic plasma membrane maintains a strong negative surface charge due to the dense arrangement of anionic phospholipid headgroups, the positively charged functional groups of Lysine and Arginine residues within CDP4 engage in rapid, high-affinity electrostatic docking. This interaction anchors the peptide firmly to the cell surface, triggering the internalization cascade. Truncation or specific point mutations within this cluster severely impair cellular uptake, which directly correlates with a loss of downstream osteogenic differentiation (Lee et al., 2019).

Biophysical profiling reveals that CDP4 possesses a low-to-moderate hydrophobic moment (*μ*_*H*_). Even though its primary sequence contains aromatic and hydrophobic elements-specifically Valine, Proline, and Tyrosine - their spatial allocation prevents the formation of perfectly segregated hydrophobic/hydrophilic faces upon secondary folding. Consequently, the peptide is defined by a linear amphipathicity rather than a spatial (helical) one, created by the charge asymmetry between the neutral N-terminal block (VNPKY) and the strongly cationic C-terminus (Schmitt and Wennemers, 2026).

In aqueous environments, circular dichroism (CD) shows that CDP4 maintains a predominantly disordered, random coil secondary conformation, typical for short, highly charged sequences. However, structural integrity is enhanced by the presence of a Proline residue, which creates a specific structural kink. This steric constraint suppresses premature, unproductive folding or aggregation, keeping the peptide in a highly flexible state that accelerates membrane translocation (Rey et al., 2023).

Furthermore, Asparagine and Glutamine residues provide critical amphipathic stabilization. This balance grants CDP4 the conformational plasticity needed to seamlessly adapt to highly contrasting microenvironments-enabling solubility in the aqueous extracellular space and cytosol, while facilitating transition through the non-polar, fatty core of the lipid bilayer (Kavyashree et al., 2026; Schmitt and Wennemers, 2026).

The cytocompatibility of CDP4 hinges on a delicate balance between its overall hydrophobicity and positive charge density. While excessive cationic charges typically cause membrane lysis in non-target eukaryotic cells, the structural configuration of CDP4 ensures highly selective affinity for target osteoprogenitor and endothelial lineages without inducing hemolysis (Kim et al., 2021). This provides a non-toxic vector for delivering intracellular signals in regenerative therapies.

#### 5.2.2 Structure - activity relationships (SAR) and intracellular signaling mechanics

The dual therapeutic efficacy of CDP4 (osteogenesis and angiogenesis) relies heavily on its sequence architecture (Pham et al., 2024; Tymińska et al., 2026a; 2026b). The core VNPKY N-terminal motif serves as the critical pharmacophore required to interface with membrane receptors, modulating the cytoskeleton and activating downstream intracellular signaling cascades. In osteoprogenitor cells (DPSCs/BMSCs), this interaction sparks the activation of mitogen-activated protein kinase (MAPK) pathways, which upregulates the transcription of runt-related transcription factor 2 (*Runx2*) and alkaline phosphatase (ALP), matching the potency of recombinant proteins like BMP-2 (Kim et al., 2021; Liao et al., 2021).

Comprehensive structure-activity relationship (SAR) data reveal stringent design constraints for the peptide. N-terminal truncation drastically downregulates or completely eradicates its osteogenic activity due to the destruction of the receptor-binding domain (Van Itallie et al., 2013). Conversely, removing the cationic C-terminal tail (ΔKQKRR) completely eliminates the peptide’s ability to penetrate the cell, rendering it biologically inert both *in vitro* and *in vivo* due to its inability to reach intracellular molecular targets (Reissmann, 2014; Schmitt and Wennemers, 2026). Furthermore, alanine scanning demonstrated that systematically replacing basic Lys and Arg residues with neutral alanine decreases any minor residual toxicity, but simultaneously drastically reduces cellular internalization rates and impairs overall biological efficacy (Madani et al., 2011).

#### 5.2.3 Proteolytic stability and degradation profile

A primary hurdle for the clinical translation of native CDP4 is its poor pharmacokinetic profile *in vivo*. Unmodified CDP4 is highly susceptible to circulating proteases, exhibiting a narrow serum half-life ( $t_{1/2}$ ) often under 30 min due to rapid clearance by plasma peptidases (Böttger et al., 2017; Cavaco et al., 2021). Its inherently unstructured, random coil status leaves the peptide backbone completely exposed, removing any steric shielding against enzymatic hydrolysis (Reveret et al., 2023) (Table 1).

**TABLE 1:** Degradation status within the proteolytic microenvironment and the resulting biochemical mechanisms and structural susceptibilities.

Proteolytic microenvironment	Degradation status	Biochemical mechanism and structural susceptibility
Trypsin	extremely rapid lysis	Trypsin selectively cleaves peptide bonds at the C-terminal side of Lys and Arg (Olsen et al., 2004). Having 5 specific cleavage sites within VNPKYKQKRR, CDP4 is rapidly degraded into inactive fragments
Serum Proteases	rapid degradation	Plentiful circulating endo/exopeptidases attack the unprotected terminal ends, yielding a short half-life (*t*1/2 < 30 min), (Böttger et al., 2017; Cavaco et al., 2021)
Thrombin	moderate susceptibility	As a selective serine protease, thrombin cleaves after Arg residues but prefers specific flanking hydrophobic domains missing in the native CDP4 sequence. It slowly cleaves the C-terminal KRR motif (Friet et al., 2026; Schmitt and Wennemers, 2026)
Matrix Metalloproteinases (MMPs)	low to moderate	MMPs show lower affinity for CDP4, as the peptide lacks the classic hydrophobic consensus cleavage patterns (such as Gly - Pro clusters found in collagen) (Böttger et al., 2017)
pH-Dependent Stability Matrix	highly variable	Acidic (pH < 5.0): Cationic charge is fully preserved, but rapid proteolysis occurs via pepsin Physiological (pH 7.4): Highly stable against chemical aggregation but enzymatically vulnerable Alkaline (pH > 8.5): Induces deprotonation of Lys residues, causing structural distortion and accelerated chemical hydrolysis (Rey et al., 2023)

Due to such rapid enzymatic clearance, *in vitro* experiments routinely require high concentrations (on the order of ∼200 µM) to sustain therapeutic activity (Nordell et al., 2026). This raises valid translational questions about achieving stable local concentrations in clinical models. Furthermore, safety screens must be extended to non-target cells (such as immune, endothelial, and chondrocyte lineages). Given the documented oncogenic pathways associated with CPNE1/3/8 over-expression and the role of nucleolin in tumor progression (Li S. et al., 2025), a rigorous, comprehensive safety profile is mandatory before clinical deployment (Shu et al., 2025; Zhang et al., 2026).

#### 5.2.4 Strategies for improving stability and biomaterial integration

To protect CDP4 from rapid degradation and enhance its therapeutic delivery, specialized chemical alterations and advanced biomaterial pairing strategies are being implemented (Figure 4):

**FIGURE 4:**
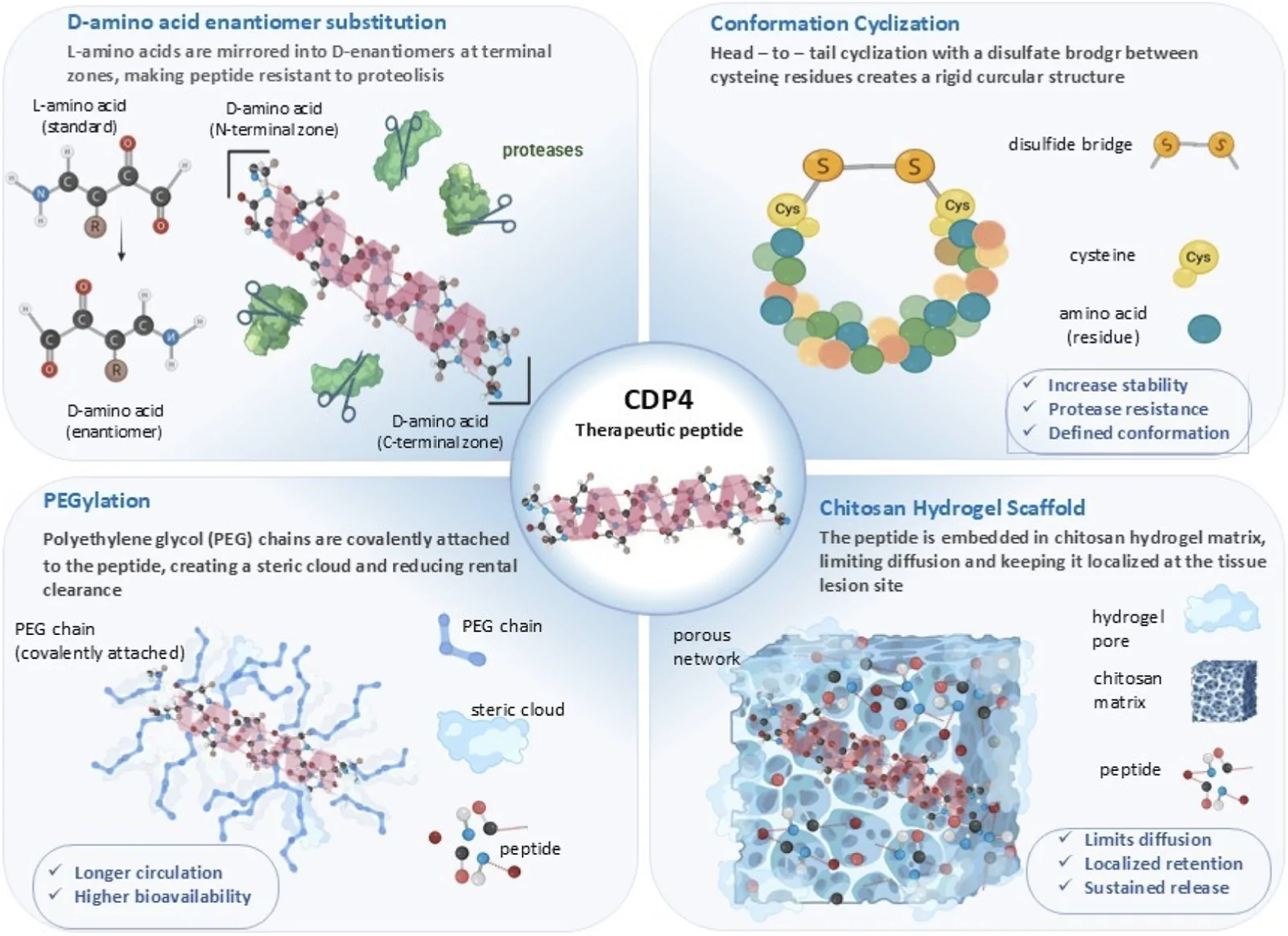
Specialized chemical modifications and advanced strategies for bonding with biomaterials. (figure by Biorender).

D-amino acid substitution: Replacing natural L-amino acids with D-enantiomers (specifically at the terminal zones) provides stereochemical resistance against endogenously active proteases (Khlaychinda et al., 2026). This shields the peptide from proteolytic detection while preserving its core osteogenic and angiogenic properties (Ruczynski et al., 2015).

Conformational cyclization: Utilizing head-to-tail cyclization or engineering intramolecular disulfide bridges via flanking Cysteine mutations restricts the peptide’s conformational flexibility (Chatterjee et al., 2008). This rigid engineering shuts down exopeptidase digestion, boosting overall plasma stability (Ruczynski et al., 2015).

PEGylation: Covalently binding polyethylene glycol (PEG) chains to the peptide introduces a strong steric barrier and expands its hydrodynamic radius (Vlieghe et al., 2010; Cavaco et al., 2021). This alteration slows down renal clearance and guards against proteolysis, though the PEG mass must be carefully balanced to prevent blocking receptor-docking kinetics.

Biomaterial Scaffolding: Groundbreaking research demonstrates that immobilizing CDP4 within natural biopolymer architectures, specifically chitosan-based hydrogels and matrices, dramatically limits peptide diffusion away from the lesion site (Kong et al., 2024; Pham et al., 2024). This biomaterial hybridization stabilizes the peptide microenvironment, prolongs its local bioavailability, and establishes a synergistic niche that accelerates tissue regeneration (Vishwanath et al., 2025).

## 6 Tissue engineering and regenerative medicine

The biological activity of CPNE7-derived peptides is strongly shaped by the physicochemical properties of the biomaterial scaffold into which they are incorporated. Parameters such as matrix stiffness, porosity, degradation rate, and the nature of peptide–matrix interactions determine local peptide availability, influence lineage commitment, and ultimately govern the quality of tissue regeneration. These factors are particularly relevant for short, highly charged peptides such as CDP4 and its engineered derivatives, whose biological performance depends on controlled release and microenvironmental cues.

The action of the CDP4 peptide in regenerative processes is strictly dependent on both its own physicochemical properties and the biomaterial in which it is embedded. CDP4 is a ten-amino-acid, hydrophilic peptide designed to easily penetrate cells due to the presence of three lysine and two arginine residues, which confer upon it the characteristics of a cell-penetrating peptide (Ruczynski et al., 2015; Lee et al., 2019). This property allows it to rapidly penetrate the cytoplasm and activate Ca^2+^- dependent pathways, which results in cytoskeletal modulation and the regulation of mesenchymal cell differentiation. At the same time, its hydrophilicity promotes rapid release from biomaterials, as confirmed by diffusion kinetics studies - approximately 50% of the peptide is released from a 4% collagen gel within the first 2 days, and up to 60% over the following 2 weeks (Lee et al., 2019).This means that CDP4 can act both in the early phase of regeneration and in the tissue remodeling phase.

Our investigations demonstrated that the functionalization of the CDP4 peptide via the addition of a flanking PEN-FFG domain (UG27) stimulates extracellular matrix (ECM) biomineralization and enhances osteogenesis by approximately 30% relative to the untreated control (Tymińska et al., 2026b). Mechanistically, this pro-osteogenic effect is mediated by a cascade involving the initiation of mitophagy, subsequent macroautophagy, and the deposition of crystalline apatite structures. Circular dichroism spectroscopy and molecular dynamics simulations confirmed that the introduction of the PEN-FFG sequence induces no steric hindrance and does not perturb the native random-coil conformation of the core CDP4 sequence. Consequently, the intrinsic biochemical affinity and metabolic accessibility of the active core are fully preserved, validating the therapeutic potential of UG27 in targeted bone tissue regeneration.

Conversely, chitosan-based matrices provide a microenvironment optimized for chondrogenic commitment. As a polycationic polysaccharide, chitosan electrostatically sequesters endogenous glycosaminoglycans (GAGs) and stabilizes cellular aggregates, thereby recapitulating the physiological signaling milieu of the cartilaginous matrix. Covalent immobilization of the CDP4 peptide onto the chitosan hydrogel significantly enhanced mesenchymal stem cell survival, focal adhesion formation, total collagen synthesis, and GAG deposition during the early stages of chondrocyte differentiation (Figure 5) (Tymińska et al., 2024).

**FIGURE 5:**
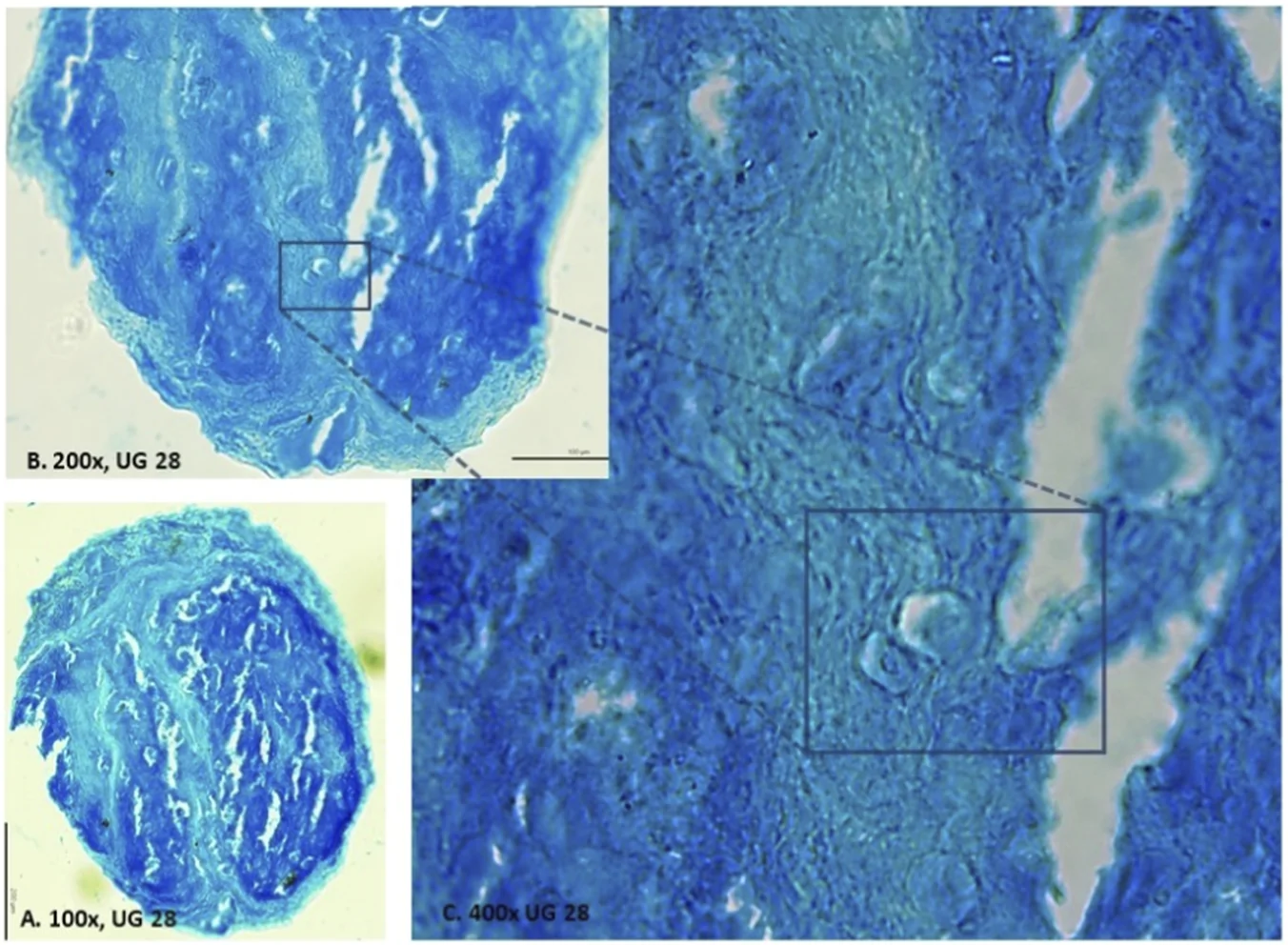
Adipose-derived mesenchymal stem cells (AD-MSCs) underwent chondrogenic differentiation following stimulation with UG28 peptide at a concentration of 10 μg/mL after 6 weeks of culture. Image of the cells after staining glycosaminoglycans (GAGs) with Alcian blue [Laboratory of Tissue Engineering and Regenerative Medicine, Division of Embryology, Department of Anatomy, Faculty of Medicine, Medical University of Gdansk (Tymińska et al., 2024)]. The photo shows isogenous groups with chondrocytes, with a magnification of **(A)**. 100x, **(B)** 200x, **(C)** 400×. The photograph was taken with a Leica inverted light microscope.

To achieve site-specific covalent immobilization, the peptide was synthesized with a terminal cysteine residue (CPLG-UG28), enabling chemoselective conjugation to maleimide-functionalized chitosan through a stable thioether linkage. This architecture provides sustained, microenvironment-responsive cargo release via the integration of the PLG-LEA linker (UG28 peptide), which serves as a substrate for matrix metalloproteinase-7 (MMP-7). Local upregulation of MMP-7 during chondrogenic remodeling results in the site-specific hydrolysis of the PLG↓LEA scissile peptide bond. The resulting liberated bioactive product consists of the elongated LEA-VNPKYKQKRR sequence. Conformational mapping and functional assays established that the residual N-terminal LEA overhang does not compromise the functional topology of the peptide; the active core retains its capacity for rapid membrane translocation and downstream Ca ^2+^ signaling activation. This peptide-mediated signaling acts synergistically with the low-stiffness, low-permeability chitosan hydrogel to effectively stabilize and maintain the chondrogenic phenotype (Tymińska et al., 2024).

The combination of both biomaterials demonstrates that CDP4 is a peptide with broad regenerative potential, and the direction of cell differentiation depends on the microenvironment in which it is placed. Collagen enhances its osteogenic activity, leading to intense mineralization and bone remodeling, while chitosan supports chondrogenesis, stabilizing cells toward a chondrogenic phenotype (Lee et al., 2019; Tymińska et al., 2024). The peptide’s properties - hydrophilicity, ability to penetrate cells, rapid release, and activation of Ca^2+^ pathways - enable it to adapt to both environments, making it a versatile modulator of regenerative processes. Understanding this interaction between the peptide and the biomaterial is crucial for designing targeted reconstructive therapies in which CDP4 can be precisely tailored to the needs of the target tissue.

An expanding body of evidence indicates that members of the copine (CPNE) family function as versatile, calcium-dependent regulators that coordinate membrane dynamics, cytoskeletal organization, and developmental signaling. Across multiple tissue types, CPNE proteins respond to fluctuations in intracellular Ca^2+^ by translocating to the plasma membrane and recruiting specific effector proteins. This rapid, Ca^2+^-dependent relocalization enables them to modulate processes such as vesicular trafficking, cell - matrix interactions, and lineage specification (Creutz et al., 1998; Khvotchev and Soloviev, 2024). Such modular activity provides a mechanistic basis for the broad yet context-dependent roles of CPNE in angiogenesis, osteogenesis, neurogenesis, and epithelial maturation (Magloire et al., 2003; Oh et al., 2015; Reinhard et al., 2016; Kim et al., 2018; Choung et al., 2019; Lee et al., 2019; Tymińska et al., 2026b; 2026a).

A major conclusion emerging from recent studies is that CPNE proteins are not passive structural components but dynamic signaling nodes. The C2 domains confer membrane affinity and Ca^2+^ sensitivity, whereas the conserved A domain mediates protein - protein interactions (Lee et al., 1995; Nalefski and Falke, 1996; Creutz et al., 1998; Tomsig et al., 2003; Khvotchev and Soloviev, 2024). This domain architecture allows CPNE to operate as adaptive modules integrating multiple developmental cues. Such functional plasticity likely explains why individual CPNE isoforms display tissue-specific activities despite sharing a similar structural organization.

A particularly notable finding is that short peptides derived from CPNE7 can reproduce selected regenerative functions of the full-length protein (Lee et al., 2020; Tymińska et al., 2024; 2026b). The CDP4 peptide exemplifies this principle, demonstrating that minimal sequence motifs can retain the capacity to stimulate endothelial repair, promote osteogenic (Butler and Ritchie, 1995; Magloire et al., 2003; Seo et al., 2017; Lee et al., 2020; Tymińska et al., 2026a) and chondrogenic differentiation (Tymińska et al., 2024), and enhance extracellular matrix deposition. These observations position CPNE proteins as an underexplored source of pro-regenerative peptide motifs with considerable translational promise.

From a biomaterials perspective, CPNE-derived peptides offer several practical advantages:

They are short and synthetically accessible via standard solid-phase methodologies, they exhibit well-defined, modular activities, they can be readily incorporated into scaffolds or delivery systems, they mimic endogenous developmental signals without requiring full-length proteins (Lee et al., 2019; Tymińska et al., 2024).

Despite these advances, several key questions remain unresolved. The effector networks recruited by individual CPNE proteins have not yet been fully delineated, and the structural determinants governing peptide activity are only beginning to be understood. Moreover, the interplay between CPNE-mediated Ca^2+^ signaling and mechanical cues within regenerating tissues remains largely unexplored. Addressing these gaps will be essential for the rational design of next-generation peptides inspired by CPNE biology.

Taken together, the structural, developmental, and peptide-engineering perspectives outlined here highlight CPNE proteins as a promising platform for the design of targeted biomaterials that leverage endogenous regenerative pathways. Continued mechanistic studies are likely to expand the repertoire of bioactive motifs with genuine therapeutic potential.

## 7 Translational challenges, GMP, safety, and clinical benchmarks

Despite encouraging preclinical evidence, CPNE7-derived peptides such as Cpne7- DP, CDP4, UG27 and UG28 must still be regarded as investigational agents requiring extensive translational validation before clinical deployment. Their advancement toward therapeutic use depends on meeting rigorous manufacturing and regulatory criteria. Scalable GMP-grade synthesis with strict endotoxin control is essential, together with validated sterilization procedures-such as gamma irradiation or sterile filtration-that preserve peptide integrity. Equally important is systematic stability testing under clinically relevant storage conditions, including lyophilization, temperature fluctuations, and humidity exposure, to ensure reproducible performance across batches.

From a regulatory perspective, CPNE7-derived peptides incorporated into collagen or chitosan scaffolds constitute combination products, necessitating coordinated evaluation of both the biomaterial and the bioactive component. This classification imposes additional requirements for quality control, biocompatibility, and long-term safety assessment.

Safety considerations remain central to translational feasibility. Because full-length CPNE7 relies on nucleolin-mediated internalization, and nucleolin is highly expressed on activated endothelial cells and numerous malignant tumors, any nucleolin-binding peptide carries a theoretical risk of stimulating angiogenesis or inadvertently supporting tumor microenvironment activity (Bugler et al., 1982; Legrand et al., 2004; Wang Y. et al., 2011; Tymińska et al., 2026b; 2026a). Furthermore, the documented oncogenic roles of CPNE1, CPNE3, and CPNE8 underscore the need for careful screening of CPNE-derived peptides for proliferative or pro-survival effects in non-target tissues. Comprehensive immunogenicity and inflammatory profiling in large animal models is required to exclude adverse immune activation and to establish long-term biosafety.

Clinical translation will also require benchmarking CPNE7-derived peptides against established regenerative biologics such as BMP-2, including comparative analyses of efficacy, safety, manufacturing cost, and risk–benefit profiles. Ultimately, long-term evaluation in non-human primate bone, cartilage, and dentin defect models will be necessary to validate functional outcomes, optimize peptide release kinetics for clinically relevant defect sizes, and determine whether these peptides can achieve durable regeneration without systemic toxicity.

## 8 Conclusion

Copines (CPNE) represent a highly dynamic, yet vastly under-explored intracellular regulatory platform, the precise elucidation of which holds the key to modern biomedical strategies. The role of these proteins as sensors coupling fluctuations in Ca ^2+^ concentration with the remodeling of cellular architecture establishes a new paradigm in the design of targeted therapies. Furthermore, the unique, modular topology of these proteins provides a ready structural template for biomimetic engineering. Successes in mapping functional CPNE domains, alongside the implementation of advanced delivery systems using smart, stimuli-responsive biomaterials, demonstrate that copine derivatives can serve as a universal foundation for a new generation of synthetic pro-regenerative agents.

## 9 Systematic literature search methodology

To provide a comprehensive, rigorous synthesis of copine biology and its translational applications in tissue engineering, a systematic search strategy was conducted across PubMed, Scopus, and Web of Science databases up to September 2026. Search queries incorporated combination strings of Boolean operators: (‘Copine’ OR ‘CPNE’ OR ‘CPNE1-9′ OR ‘CPNE7’) AND (‘calcium sensor’ OR ‘tissue development’ OR ‘synaptic plasticity’ OR ‘odontogenesis’ OR ‘osteogenesis’ OR ‘cancer’ OR ‘CDP4’ OR ‘Cpne7-DP’ OR ‘biomaterial’ OR ‘tissue engineering’). Inclusion criteria comprised peer-reviewed original research articles, structural biology papers, transcriptomic profiles, and biomaterial functionalization studies.

## Glossary

AD-MSCs: Adipose-derived mesenchymal stem cells
AKT (PKB): Protein kinase B
ALP: Alkaline phosphatase
AMP: Adenosine monophosphate
ATP: Adenosine triphosphate
BMP-2: Recombinant human bone morphogenetic protein-2
BMSCs: Bone marrow mesenchymal stem cells
BON1/2/3: BONZAI 1, 2, and three proteins (in *Arabidopsis thaliana*)
cDNA: Complementary deoxyribonucleic acid
ccRCC: Clear cell renal cell carcinoma
CD: Circular dichroism spectroscopy
CDP: Copine-derived peptide
CPLG-UG28: Cysteine-containing matrix metalloproteinase-sensitive peptide derivative
CPNE: Copine protein family
CPNE1–CPNE9: Copine paralogs 1 through 9
Cpne7-DP: Copine 7-derived peptide
CPP: Cell-penetrating peptide
Dmp1 (*Dmp*1): Dentin matrix acidic phosphoprotein 1 (protein/*gene*)
DPSCs: Dental pulp stem cells
Dspp (*Dspp*): Dentin sialophosphoprotein (protein/*gene*)
ECM: Extracellular matrix
EDTA: Ethylenediaminetetraacetic acid
EGFR: Epidermal growth factor receptor
ERK: Extracellular signal-regulated kinase
FAK: Focal adhesion kinase
Fmoc: 9-Fluorenylmethoxycarbonyl protecting group
GAGs: Glycosaminoglycans
GLUT1: Glucose transporter 1
GMP: Good Manufacturing Practice
HK2: Hexokinase 2
Ift88 (*Ift88*): Intraflagellar transport protein 88 (protein/*gene*)
IGF-1: Insulin-like growth factor 1
Kif3a (*Kif3a*): Kinesin family member 3A (protein/*gene*)
LIMK1: LIM kinase 1
LPS: Lipopolysaccharide
LTP: Long-term potentiation
MAPK: Mitogen-activated protein kinase
MIDAS: Metal-ion-dependent adhesion site motif
MMP-7: Matrix metalloproteinase-7
mTOR: Mechanistic target of rapamycin
NCL: Nucleolin receptor
Nestin (*Nestin*): Nestin neurofilament protein (protein/*gene*)
NMDA: N-methyl-D-aspartate receptor
NSCLC: Non-Small Cell Lung Cancer
PAK: P21-activated kinase
PBS: Phosphate-buffered saline
PEG: Polyethylene glycol
PI3K: Phosphoinositide 3-kinase
PLG-LEA: Matrix metalloproteinase-7 cleavable scissile peptide linker
Rac1: Ras-related C3 botulinum toxin substrate 1
Runx2 (*Runx2*): Runt-related transcription factor 2 (protein/*gene*)
SAR: Structure–Activity Relationships
STAT3: Signal transducer and activator of transcription three
**t**_**1/2**_: Serum half-life
TFA: Trifluoroacetic acid
TNBC: Triple-negative breast cancer
UV: ultraviolet spectroscopy
vWA: von Willebrand factor type A domain
***μ***_***H***_: Hydrophobic moment
